# Supplementary material for: Transcriptome analysis of diploid and triploid Populus tomentosa
Source: PeerJ. 2020 Oct 28;8:e10204. doi: 10.7717/peerj.10204 (PMC7602689; doi:10.7717/peerj.10204)
Supplement: Supplemental Information 1 [file peerj-08-10204-s001.docx]

**Supplemental** Table.1 Quality index of reads aFTer filtration.

| Sample  name | Raw Reads (M) | Clean  Reads  (M) | Total Clean Bases (Gb) | Clean Reads Q30 (%) | Clean Reads Ratio (%) | GC Content  (%) |
| --- | --- | --- | --- | --- | --- | --- |
| D1 | 50.83 | 43.73 | 6.56 | 88.95 | 86.04 | 42.12 |
| D2 | 49.08 | 42.46 | 6.37 | 88.90 | 86.52 | 42.19 |
| D3 | 47.33 | 42.47 | 6.37 | 89.05 | 89.73 | 42.38 |
| T1 | 49.08 | 43.46 | 6.52 | 88.84 | 88.55 | 42.28 |
| T2 | 50.83 | 43.64 | 6.55 | 89.43 | 85.85 | 42.19 |
| T3 | 49.08 | 42.6 | 6.39 | 88.85 | 86.79 | 42.25 |

Note: D1-D3: diploid *P. tomentosa*; T1-T3: triploid *P. tomentosa.*

**Supplemental** Table.2 Statistics of reference genome alignment results.

| Sample | Clean Reads(M) | Total Mapping(%) | Uniquely Mapping(%) |
| --- | --- | --- | --- |
| D1 | 43.73 | 78.63 % | 20.58 % |
| D2 | 42.46 | 78.46 % | 20.34 % |
| D3 | 42.47 | 81.3 % | 21.44 % |
| T1 | 43.46 | 80.47 % | 20.75 % |
| T2 | 43.64 | 78.06 % | 19.56 % |
| T3 | 42.6 | 79.14 % | 20.2 % |

Note: D1-D3: diploid *P. tomentosa*; T1-T3: triploid *P. tomentosa.*

**Supplemental** Table.3 Functional annotation results of unigenes.

| Values | Total  Number of unigenes | NR | NT | Swissprot | KEGG | KOG | Pfam | GO | Overall |
| --- | --- | --- | --- | --- | --- | --- | --- | --- | --- |
| Number | 77,006 | 66,554 | 71,647 | 50,143 | 52,404 | 51,971 | 50,881 | 50,210 | 72,240 |
| Percentage | 100% | 86.43% | 93.04% | 65.12% | 68.05% | 67.49% | 66.07% | 65.20% | 93.81% |
